# Investigation of the essential role of platelet-tumor cell interactions in metastasis progression using an agent-based model

**DOI:** 10.1186/1742-4682-11-17

**Published:** 2014-04-12

**Authors:** Abhineet Uppal, Sean C Wightman, Sabha Ganai, Ralph R Weichselbaum, Gary An

**Affiliations:** 1 Department of Surgery, University of Chicago, 5841 South Maryland Avenue, MC 5029, Chicago, IL 60637, USA; 2 Department of Surgery, Simmons Cancer Institute at SIU, 315 W Carpenter, Springfield, IL 62702, USA; 3 Department of Radiation Oncology, The University of Chicago Medicine, 5841 S. Maryland Avenue, MC 9006, Chicago, IL 60637, USA; 4 Department of Surgery, The University of Chicago Medicine, 5841 S. Maryland Avenue, MC 5094 S-032, Chicago, IL 60637, USA

**Keywords:** Agent-based modeling, Computational modeling, Metastasis

## Abstract

**Background:**

Metastatic tumors are a major source of morbidity and mortality for most cancers. Interaction of circulating tumor cells with endothelium, platelets and neutrophils play an important role in the early stages of metastasis formation. These complex dynamics have proven difficult to study in experimental models. Prior computational models of metastases have focused on tumor cell growth in a host environment, or prediction of metastasis formation from clinical data. We used agent-based modeling (ABM) to dynamically represent hypotheses of essential steps involved in circulating tumor cell adhesion and interaction with other circulating cells, examine their functional constraints, and predict effects of inhibiting specific mechanisms.

**Methods:**

We developed an ABM of Early Metastasis (ABMEM), a descriptive semi-mechanistic model that replicates experimentally observed behaviors of populations of circulating tumor cells, neutrophils, platelets and endothelial cells while incorporating representations of known surface receptor, autocrine and paracrine interactions. Essential downstream cellular processes were incorporated to simulate activation in response to stimuli, and calibrated with experimental data. The ABMEM was used to identify potential points of interdiction through examination of dynamic outcomes such as rate of tumor cell binding after inhibition of specific platelet or tumor receptors.

**Results:**

The ABMEM reproduced experimental data concerning neutrophil rolling over endothelial cells, inflammation-induced binding between neutrophils and platelets, and tumor cell interactions with these cells. Simulated platelet inhibition with anti-platelet drugs produced unstable aggregates with frequent detachment and re-binding. The ABMEM replicates findings from experimental models of circulating tumor cell adhesion, and suggests platelets play a critical role in this pre-requisite for metastasis formation. Similar effects were observed with inhibition of tumor integrin αV/β3. These findings suggest that anti-platelet or anti-integrin therapies may decrease metastasis by preventing stable circulating tumor cell adhesion.

**Conclusion:**

Circulating tumor cell adhesion is a complex, dynamic process involving multiple cell-cell interactions. The ABMEM successfully captures the essential interactions necessary for this process, and allows for *in-silico* iterative characterization and invalidation of proposed hypotheses regarding this process in conjunction with *in-vitro* and *in-vivo* models. Our results suggest that anti-platelet therapies and anti-integrin therapies may play a promising role in inhibiting metastasis formation.

## Introduction

Metastatic disease presents a significant burden in cancer treatment and could be considered perhaps the most critical aspect of oncology in terms of control of established cancers. Currently, strategies for treatment of metastatic cancer focus on limiting the growth and further extension of metastatic disease, either by removal surgically or using chemotherapy or radiation therapy to eradicate or limit existing sites. Given the clinical challenges of dealing with established metastases, there is an increasing focus in limiting the generation of metastatic foci through better understanding of the processes leading from a primary tumor to metastasis [1]. It is well recognized that the process of metastasis is a complex, multi-step process involving tumor cell interactions with the host’s vascular and immune systems at multiple levels: a non-comprehensive list of the steps required to develop a metastasis include tumor shedding from the primary cancer, the lifecycle/dynamics of circulating tumor cells and the process of metastasis colonization. (Reviewed in [2-9]). While each of these processes are the subject of intense study, it clear that there is a defined sequence of events with dependencies and consequences between levels of processes. This suggests that being able to effectively modulate the generation of metastases requires an integrated view of the multiple events involved. Challenges to an integrated view of the formation of a metastasis include the rarity of these events, the variability of tumor metastatic and growth potential, and the heterogeneity of host organs for metastasis [1,2,4,10-14].

We focus on the vital step between the conditions leading to the generation of circulating tumor cells from the primary tumor and the process of invasion of those circulating tumor cells: the initial adhesion of shed and circulating tumor cells to their potential target tissue beds. Though millions of tumor cells are detectable in the circulation of patients with primary tumors, very few have the ability to successfully adhere, invade and proliferate [4,13]. Coagulation events are known to play a significant role, with one important recognized interaction being the role of platelet activation in circulating tumor cells adhesion to the endothelium [15-18]. Given the relationship between coagulation and inflammation, we posit that there are potential down-stream effects from the initial tumor-coagulation-endothelial interactions on endothelial permeability, tumor cell killing and the host tissue inflammatory/immune milieu, with consequent influences on the establishment of metastases. Additionally, there exists some evidence that interdiction at the early events of tumor cell adhesion has an effect on the establishment of metastases. Notably, aspirin, a potent inhibitor of platelet activation, has been found to reduce the incidence of metastasis in patients [10,14]. In addition, inhibition of tumor cell expression of integrin αV/β3 has been shown to decrease melanoma, colorectal and breast cancer cell adhesion to endothelium and platelets, resulting in decreased number of metastases [16,19-21]. The characterization of the establishment of metastases as a cascading series of events prompts our focus on the most upstream interactions associated with the establishment of metastases in order to provide a functional context for complementary investigations in the downstream processes of metastasis formation.

However, there are significant challenges to the experimental study of the establishment of metastases from circulating tumor cells. In particular, longitudinal observation of single cells in circulation to metastasis formation is technically difficult in animal models, and impossible in humans. This results in a host of potential variables extremely difficult to account for in experimental models. Identifying which of myriad genetic variations is responsible for a phenotype is resource intensive and potentially intractable without use of new methodologies.

These challenges can potentially be met by dynamic computational modeling to represent and interrogate relevant variables of existing mechanistic knowledge within a functional context of complex cellular interactions [22-25]. Dynamic modeling allows for variables to be adjusted *in silico* and resulting behaviors observed with more ease and at a higher degree of spatial and temporal resolution than can be achieved with standard biological models. This allows for more rapid consideration of the plausibility of potential mechanisms, discarding those clearly not correct and allowing experimental resources to be focused on the most plausible hypotheses [23,26-29].

One method used for computational dynamic knowledge representation is agent-based modeling [30-35]. Agent-based models (ABMs) can be used to simulate complex interactions as they are made of populations of computational agents, mimicking cells, that follow programmed rules, in parallel, that regulate their interaction with the environment and one another. Variability in response to certain inputs and production of outputs simulates the diversity of cellular behavior in a complex environment. The effect of altering specific variables on the complex dynamics generated can be examined in simulation runs. The outputs of experiments are provided continuously in a visual format that can be compared to biological experiments.

We have developed a descriptive, first-generation agent-based computational model that incorporates observed cellular behaviors and phenomenon in order to simulate the basic dynamics of circulating tumor cell adhesion in the context of endothelial, neutrophil and platelet interactions: the Agent-Based Model of early metastasis (ABMEM). Circulating tumor cell adhesion involves recruitment of neutrophils and platelets, multiple cell-cell interactions, initiation of cellular processes by cytokines, and activation of the coagulation cascade. These processes culminate in the stable binding of tumor cells to endothelial cells, a necessary precursor for subsequent tumor cell invasion into the host organ. Though not a predictive model, the ABMEM allows us to propose which mechanisms are essential for stable tumor cell adhesion and thus may represent potential therapeutic targets for anti-metastasis therapy.

## Results

### Overview of the Agent-Based Model of Early Metastasis (ABMEM)

The ABMEM integrates currently known mechanistic knowledge observed in published biological models of tumor, neutrophil, platelet and endothelial interactions (see Table 1 and the Materials and Methods for a list of components of the model). Development of the ABMEM was performed in an iterative manner, with successive layers of validation in regards to known behaviors, a procedure referred to as the Iterative Refinement Protocol [19,28,36-39]. Initial iterations of the ABMEM focused on producing *face validity*, the ability of the model to behave in a biologically plausible fashion [40,41]. Subsequent iterations emphasized *experimental validity* through addition of mechanistic details if the existing model is unable to reproduce the behaviors of interest observed in experimental systems [42,43].

**Table 1:** Key Molecular Pathways Represented in the ABMEM

**Protein**	**Function in ABM**
Integrin αV/β3	Expressed by tumor cells, binds activated platelet GpIIB/IIIa and activated PMN Mac-1 (αM/β2)
Mac-1 and LFA-1	Expressed by PMNs, binds platelet GpIbα and tumor cell Integrin αV/β3
N-cadherin	Expressed by PMNs, initiates rolling on endothelial cells.
L-selectin	Expressed by PMNs, initiates rolling on endothelial cells.
GpIα	Expressed by platelets, binds PMN αM/β2, activated by GpIIb/IIIa
GpIIb/IIIa	Expressed by platelets, binds endothelial cells and tumor cells.
P-selectin	Expressed by platelets, initiates binding to tumor cells, PMNs and endothelial cells.
Thrombin	Generated by platelets, PMNs and tumor cells, diffuses through world and activates platelets, PMNs and endothelial cells.
Thromboxane (TXA)	Generated by activated platelets, activates GpIIb/IIIa
Adenosine Diphosphate (ADP)	Generated by platelets, diffuses and activates GpIIb/IIIa and P-selectin
Interleukin-8 (IL-8)	Generated by tumor cells, activated PMNs and activated endothelial cells, diffuses through world and activates other cells.
Nitric Oxide (NO)	Generated by endothelial cells, inhibits PMN ROS production and inhibits endothelial cell activation.

**Figure 1:**
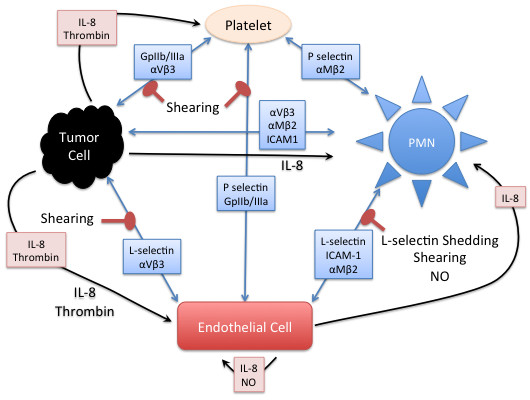
**Overall schematic of interactions between tumor cells, neutrophils, platelets and endothelial cells within the ABMEM.** Blue arrows represent binding interactions between cell surface receptors. Black arrows represent diffusion of released mediators. Red arrows represent inhibition of a cell-cell interaction. PMN = polymorphonuclear leukocyte (neutrophil), IL-8 = interleukin-8, NO = nitric oxide.

**Figure 2:**
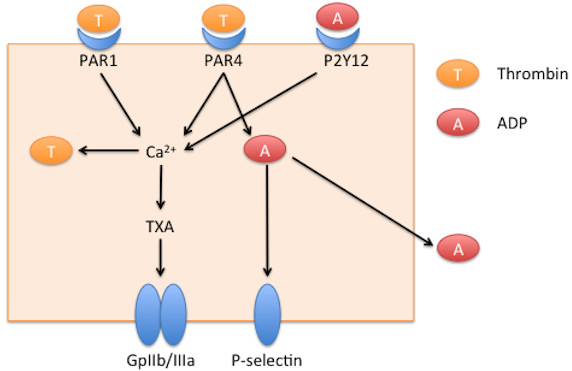
**Mechanisms of activation incorporated into the platelet agents of the ABMEM.** Extracellular Thrombin (T) binds to Protease-activated receptor (PAR1) and Protease-activated receptor 4 (PAR4) on the platelet agent, inducing the thromboxane (TXA) pathway in a calcium dependent manner to cause the platelet to activate Integrin GpIIb/IIIa. PAR4 also activates intracellular adenosine diphosphate (ADP, A) production. Intracellular ADP induces platelet P-selectin expression. ADP is also released into the extracellular environment and acts in an autocrine and paracrine fashion by binding to Purinergic receptor P2Y (P2Y12) on the platelet, contributing to calcium-mediated expression of the Integrin GpIIb/IIIa.

**Figure 3:**
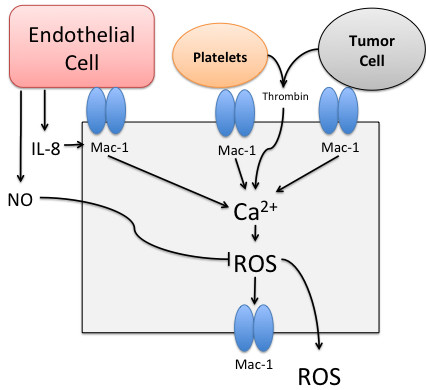
**Mechanisms of activation incorporated into the neutrophil agents of the ABMEM.** Endothelial cells, Platelets and Tumor cells bind to activated Macrophage Antigen 1 (Mac-1). Interleukin 8 (IL-8) released by endothelial cells induces further Mac-1 activation. Thrombin (T) released by Platelets or Tumor cells, and activation of Mac-1 induces reactive oxygen species (ROS) production through a calcium dependent manner, which in turn induces further Mac-1 activation. ROS are also released into the extra-cellular environment to interact with other agents. Nitric oxide released by endothelial cells inhibits ROS production in neutrophils. IL-8: Interleukin-8. ROS – reactive oxygen species. NO – nitric oxide.

The computational agents represent the four cell types experimentally shown to be involved in tumor cell adhesion: neutrophils, platelets, endothelial cells and the tumor cells themselves. Figure 1 displays the interactions between cell types included in the ABMEM. Agents have internal state variables representing molecular components of cells: receptors, cell-surface proteins, genes, and signaling molecules. The pathways modeled were abstractly represented with logic-based, algebraic rules: these are demonstrated in Figure 2 for platelet agents and Figure 3 for neutrophil agents. The level of available active surface receptors and ligands were represented as state variables, and a threshold necessary for activation was fit for each variable. More details of the ABMEM can be seen in *Materials and Methods*. Following initial development and calibration we used the ABMEM to predict the effects of inhibiting the different adhesion mechanisms on tumor cell adhesion in the terms of dependencies on neutrophil, platelet and endothelial interactions.

### Simulation experiments

#### Calibration and initial validation of neutrophil, platelet and endothelial cell dynamics

The ABMEM was calibrated by confirming that the dynamics between neutrophils, platelets and endothelial cells behaved in a plausible fashion by binding and activating in expected manners in response to endothelial inflammation, thrombin generation and cytokine production. This step establishes *face validity* of the ABMEM, i.e. establishing that the model performs in an intuitively plausible fashion as compared to existing real world reference systems [41,44]. Rates for signal molecule production and diffusion, and adhesion molecule binding levels for cell-cell interactions were adjusted to fit neutrophil rolling, endothelial binding, platelet binding and thrombin generation in appropriate timescales as observed *in vitro.* The desired plausible behavioral criteria included: 1) the maintenance of a dynamic, steady state of neutrophil rolling and platelet inactivation on non-inflamed endothelium [23,27,45]; 2) neutrophil and platelet activation in presence of inflamed endothelium [33,39,40,46,47]; 3) inhibition of platelet activation with inhibition of thromboxane or adenosine diphosphate (ADP) receptors [44,48].

**Figure 4:**
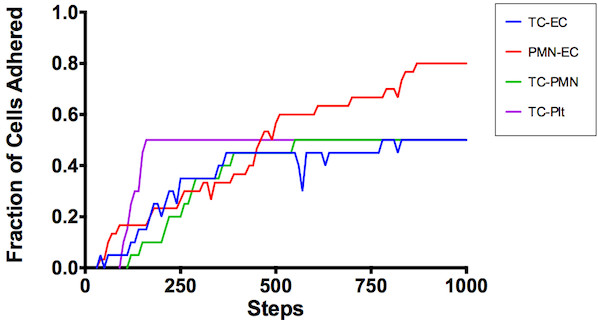
**Cell Interactions Over Time.** Representative plot of fraction of cells adhered to another cell during a model run. Tumor cells bind with platelets (TC-Plt, purple) initially, followed by adhesion to endothelial cells (TC-EC, blue) and circulating or rolling neutrophils (TC-PMN, green). Rolling neutrophils simultaneously adhere with endothelial cells (PMN-EC, red). Stable tumor cell adhesion is achieved after 750 steps.

The baseline ABMEM calibration of platelet and neutrophil behavior was followed by the addition of tumor cell agents to the model. Further heuristic tuning of tumor cell adhesion thresholds and signaling molecule production was performed to achieve similarity to interaction steps observed *in vitro* and *in vivo*[40,49-51]*.* This included circulating tumor cell adhesion to activated neutrophils and platelets, initial adhesion of tumor cells to the endothelium directly or through these mechanisms, and stable binding over time (see Additional file 1 for similarity assessments). Figure 4 demonstrates the interactions between cell types over the course of a simulation run.

### Simulation of platelet inhibition

**Figure 5:**
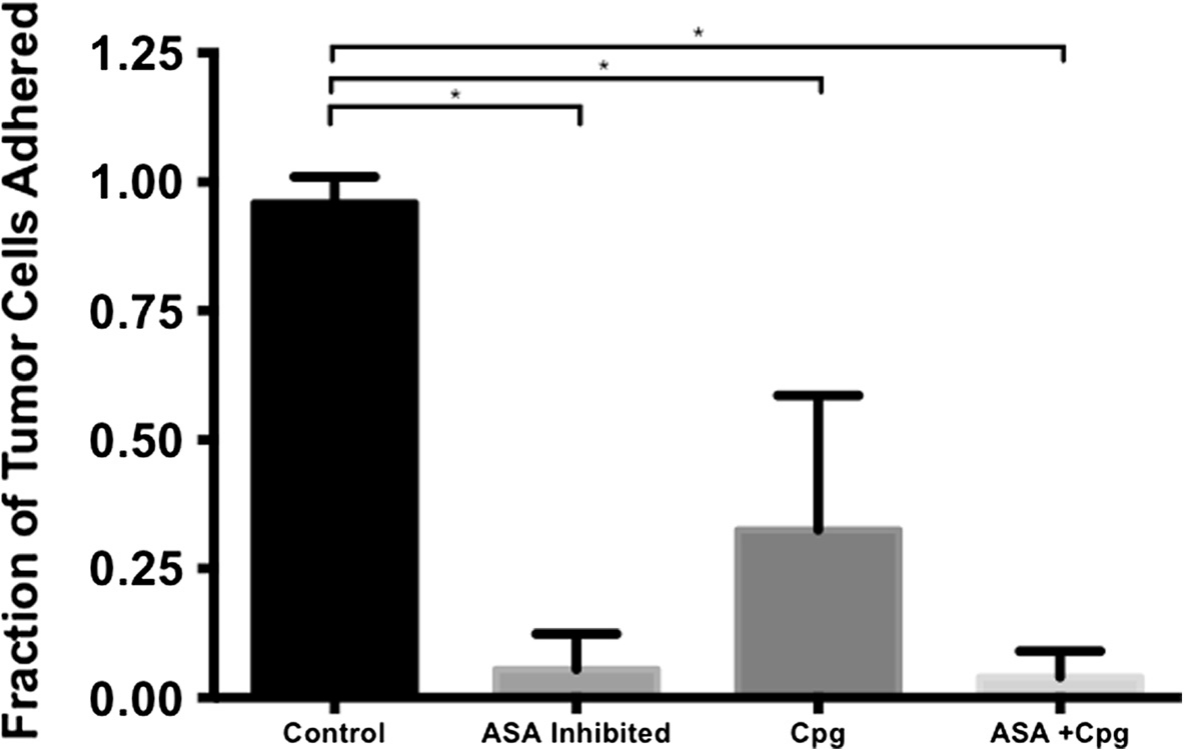
**Effect of thromboxane inhibition, ADP inhibition and combined inhibition on number of tumor cells adherent to endothelial cells.** Both ASA – Aspirin (thromboxane inhibitor) and Cpg – clopidogrel (P2Y12 ADP receptor inhibitor) individually and in combination lead to statistically significant reduction in tumor cell-endothelial cell binding. *, p <0.05.

The next simulations were performed to examine the effect of platelet inhibition on the observed dynamics of tumor cell adhesion. Specifically, we hypothesized that platelet inhibition would destabilize tumor cell binding to the endothelium, a pre-requisite step for metastatic colonization of a host organ. Two different types of platelet inhibition were examined, each corresponding to a currently clinically available anti-platelet drug: inhibition of thromboxane by aspirin and inhibition of platelet ADP receptors by clopidorel. These experiments were run for 1,000 iterations, representing a time period of approximately one hour, corresponding with the time-course of in-vitro adhesion and invasion observed in multiple tumor types [21,28,50,52-54]. Simulations (n = 20 per condition) were run to simulate the baseline platelet activity, inhibition of thromboxane by aspirin, inhibition of ADP receptors by clopidogrel and inhibition of both pathways. Outcome measures were the percentage of tumor cells stably adhered to the endothelium at the end of each run (Figure 5). Simulation experiments with baseline platelets resulted in 95 ± 5% tumor adhesion vs. 5.5 ± 7% (p < 0.001) with thromboxane inhibition, 32 ± 26% (p < 0.05) with ADP receptor inhibition and 4 ± 5% (p < 0.001) with combined inhibition. Though both ADP receptor inhibition and thromboxane inhibition prevented platelet adhesion in the presence of only neutrophils and inflamed endothelium, thromboxane inhibition alone had significantly more effect on tumor cell adhesion. The inability to maintain integrin GpIIb activation through thromboxane resulted in failure of platelets to stably adhere to tumor cells and endothelium. In contrast, inhibition of the less stable P-selectin bond was partially compensated by binding via activated integrin GpIIb. These results suggest that aspirin may have a strong anti-metastatic effect through inhibition of stable tumor cell binding to activated platelets, while clopidogrel may have a smaller effect.

### Evaluating the role of integrins: simulation of αV/β3 inhibition

**Figure 6:**
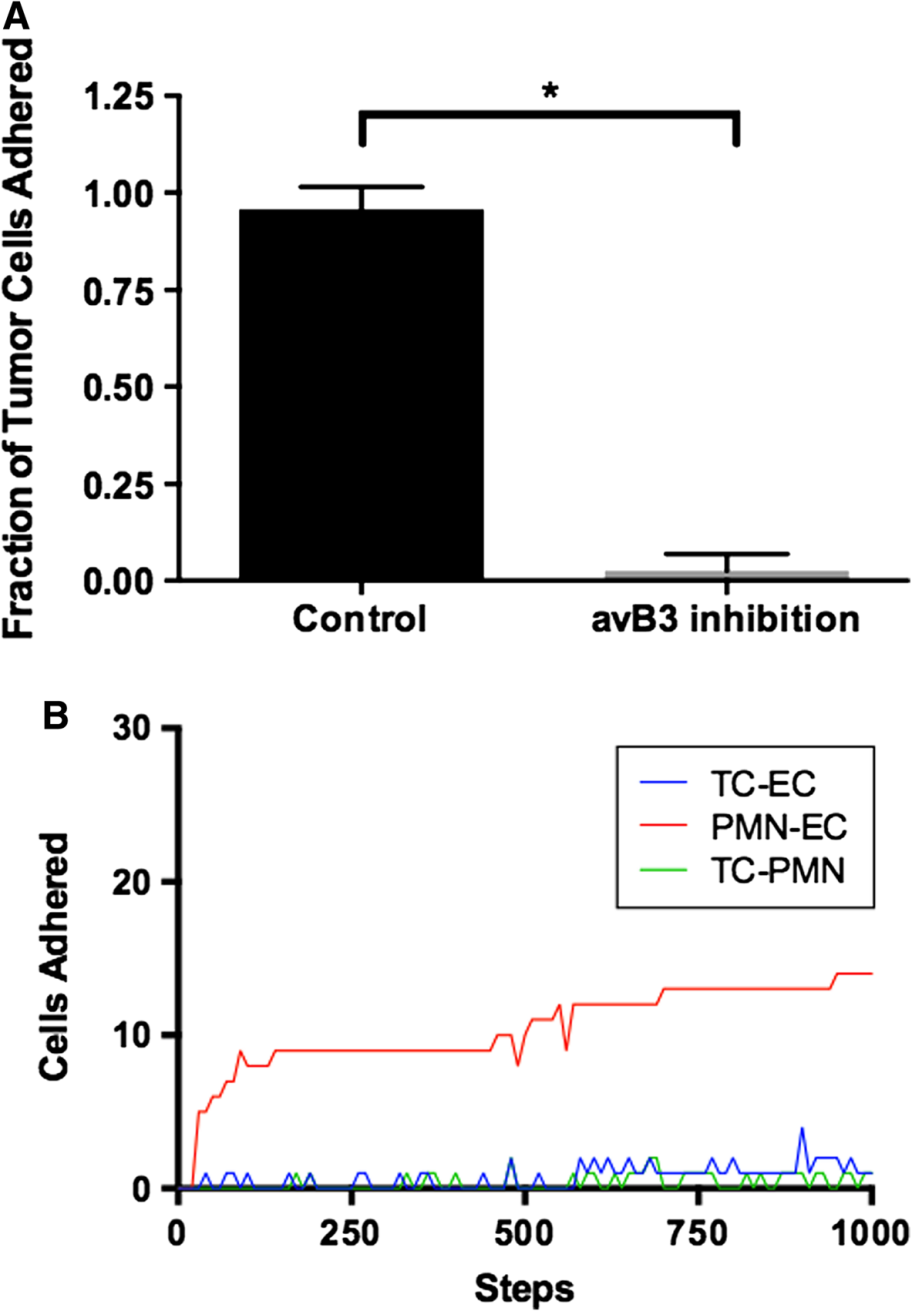
**Effect of inhibition of tumor cell Integrin αν/β3.** Panel **A** demonstrates that inhibition of tumor cell Integrin αν/β3 reduces the fraction of circulating tumor cells bound to endothelial cells. aVB3 – Integrin αν/β3. *p <0.05. Panel **B** demonstrates that that this is due to a reduction in stable binding of circulating tumor cells to neutrophils (TC-PMN, Green) and tumor cells to endothelial cells (TC-EC, Blue), with neutrophil to endothelial cell binding (PMN-EC, Red) unaffected.

Another mechanism of tumor metastasis inhibition identified in animal models is targeting of the integrin αV/β3, which is able to bind surface receptors of both platelets and neutrophils [21,32,55,56]. Monoclonal antibodies to integrin αV/β3 have been used in clinical trials to improve progression-free survival of glioblastoma purportedly through inhibition of angiogenesis [1,13,40,57-60]. Its efficacy against established metastatic disease, however, has been limited [61,62]. We hypothesize another potential mechanism of integrin αV/β3 blockade in anti-metastastis therapy: preventing circulating tumor cell adhesion to neutrophils and platelets. As the interactions between tumor cells and these cells promote stable endothelium adhesion, we hypothesized that inhibiting these bonds would attenuate the subsequent adherence of the tumor cell complex to the endothelium. Simulations were performed where integrin αV/β3 was fully inhibited to mimic the effects of the potent monoclonal antibodies used in animal and clinical studies [63,64]. These experiments were run for 1,000 steps to simulate a time course of 1 hour. Outcomes measured were stable tumor cell adhesion to endothelium and number of platelets bound to individual tumor cells. Varying degrees of inhibition of αν/β3 were examined, and 20 runs were performed for each condition. The stable binding of tumor cells was almost entirely inhibited by αV/β3 integrin suppression, though tumor cells were able to intermittently bind to PMNs through selectins (Figure 6). These simulations indicate that inhibition of αV/β3 may play an earlier role in prevention of metastasis formation than currently attempted in clinical trials by inhibiting stable circulating tumor cell adhesion.

## Discussion

### Overview of tumor cell adhesion in the context of metastasis

The formation of tumor metastasis is a complex process involving multiple interactions between tumor cells and the potential target tissue environment. Each of these steps presents an opportunity for interdiction of tumor cells progressing towards metastasis. Tumor cell adhesion to the vasculature of distant organs is an essential step in this process that exemplifies the co-opting of local tissue factors in a fashion that promotes metastasis. It involves multiple cell types and mechanisms, all of which may play a role in the ability of a given tumor cell adhering to the endothelium for sufficient time to begin the next step of intravasation. This presents a rich field of potential therapeutic targets that may curtail metastatic dissemination before tumor cells are able to colonize distant organs.

Tumor cell adhesion involves binding to endothelial cells directly and indirectly via platelets and neutrophils. Tumor cells of various primary histologies are able to activate and bind directly to endothelial cells via integrins [40,57,59,60]. Further studies showed that circulating tumor cells are able to activate and adhere to neutrophils through integrin interactions [54,65-67]. Inhibition of neutrophil activation and integrin expression results in decreased tumor cell binding to neutrophils and subsequent endothelial adhesion within the microvasculature [40,49,50]. In addition to neutrophil binding, circulating tumor cell adhesion and subsequent metastasis formation is significantly enhanced by platelet binding through integrins [16,18,68]. Inhibition of this interaction results in decreased stable adhesion to endothelium [2-9]. In addition to direct binding, tumor cells can activate platelets or neutrophils through paracrine mechanisms such as interleukin-8 (IL-8) secretion, matrix metalloproteinase secretion and thrombin induction [50,59,69-71]. These pathways represent the major known mechanisms through which tumor cells are able to stably adhere to vasculature, an essential step in the formation of hematogenous metastases. A more detailed understanding of the interplay between these mechanisms is essential for the development of therapies to limit metastatic progression. However, the complexity of this system and its multiple redundancies limits the ability of traditional reductionist biological experiments to predict downstream effects of a perturbation of a single mechanism.

Complex, dynamic systems such as circulating tumor cell adhesion can be explored through the use of computer models. Incorporating these models with traditional experimentation allows for hypotheses to be discarded or advanced for further examination in a less resource-intensive manner. Significant efforts have been made in computational modeling the multiple steps of tumor growth, with the majority of efforts examining the local invasion and growth seen in primary tumors [3,6-9]. Computational modeling of metastases has examined cell population dynamics based on a combination of proliferation, mutation and metastasis rates derived from clinical data to predict the number and time distribution of metastases from primary tumors, [72-76]. Others use mathematical models of cell growth and specific biologic processes such as matrix modeling and migration to predict metastasis growth over time [77-80]. Agent-based modeling of metastases has been employed to characterize the selective forces involved in the generation of circulating, potentially metastatic tumor cells [81,82], implicate the role of host immunity in the generation of satellite metastases [1,2,4,10-14], and examine the interactions of metastatic tumor cells with the host immune system for optimizing tumor vaccine delivery [15-18]. Other models of individual tumor cell interactions with host environment, employing knowledge of biomechanics and enzyme kinetics in a system of differential equations that describe integrin interactions and tumor cell growth patterns [83,84]. In addition, several models of single circulating cell adhesion to vasculature have been developed incorporating biomechanic principles, fluid dynamics and knowledge of integrin activation (Reviewed in [22-25]). However, these models were designed to examine single cell adhesion, not the complex processes of tumor cell interaction with other cell types. Given the multiple interactions shown to be necessary for tumor cell adhesion, a computational model incorporating these various interactions can lead to a more detailed understanding of these early adhesion events.

This ABMEM is the first computational model to examine the multiple cell-cell interactions specifically related to adhesion of circulating tumor cells to vascular endothelium. This allows for an examination of population dynamics not possible with current single-cell models biomechanical models. Incorporation of intra-cellular pathways allows for a higher-resolution understanding of the functional signaling and receptor-binding events leading to stable tumor cell adhesion. The ABMEM, as an ABM, has a modular structure, both with respect to the agents/cells included, as well as the agents’ rules. This modular design allows for the addition of further cell types (as was done with the addition of tumor cells to the ABMEM) or molecular mechanisms as necessary to validate the model against prior experimental findings. This property of ABMs is consistent with the general iterative refinement protocol described in the development of dynamic computational models [23,26-29]. This approach may promote a better understanding of the essential underlying biology by both limiting the initial set of components represented by the model and identifying when a particular model becomes insufficient to represent desired features and phenotypes of the real world system under investigation. Used in this adjunctive fashion, as a means of dynamic knowledge representation, models like the ABMEM can assist assessing the sufficiency of existing mechanistic hypotheses and aid in the generation of new hypotheses that may lead to the development of novel therapies to mitigate metastatic spread.

The ABMEM successfully represents the key mechanisms involved in tumor cell adhesion within the context of neutrophil and platelet interactions. Simulations of the inhibition of key platelet activation pathways support the concept that they may be necessary for stable tumor adhesion [16,68]. The inhibition of thromboxane by aspirin resulted in significant inhibition of tumor cell adhesion by preventing activation of GpIIb/IIIa and P-selectin. This suggests that aspirin may reduce the formation of metastases in distant organs. These findings are supported by *in vivo* models of hepatocellular carcinoma and colon adenocarcinoma [85,86]. Though thromboxane has many effects on platelets, integrin GpIIb/IIIa inhibition as modeled in the ABMEM is supported as a mechanism of reducing platelet adhesion to tumor cells, and thus decreased metastasis formation [31,33-35]. The anti-metastastic effect of aspirin is also observed in retrospective reviews of randomized controlled trials, which revealed that aspirin was a predictor of decreased metastastic burden and subsequent death in patients with primary adenocarcinomas [10,12,14]. This suggests that therapies directed towards inhibition of platelet GpIIb/IIIa activation via thromboxane may potentially reduce metastatic spread through inhibition of adhesion.

The ABMEM also revealed clopidogrel as another potential inhibitor of tumor adhesion, though its mechanism of GpIIb/IIIa and P-selectin activation is dependent on calcium production and thus is upstream of thromboxane inhibition. This novel finding has not been described by *in vivo* models or clinical data, but these results from the ABMEM simulations suggest that a retrospective review of metastatic disease in the relatively large number of patients receiving clopidogrel post-cardiac and vascular procedures might be fruitful. Additionally, the combined effect of aspirin and clopidigrel did not significantly further reduce tumor cell adhesion in the ABMEM compared to either agent alone, as a portion of tumor cells were able to adhere to the endothelium without platelet binding. As with the effect of clopidogrel alone, this is a novel finding that may be subjected to experimental testing in the future.

The ABMEM also identified direct inhibition of integrin αν/β3 expressed by tumor cells themselves as potential mechanism of tumor cell adhesion, even in the presence of platelets. This resulted in intermittent binding of tumor cells to other cells through selectins, but failure of stable integrin-based bonds to form. As noted with combined anti-platelet inhibition, a small fraction of tumor cells were still able to stably adhere to the endothelium. This is supported by *in vivo* models of colon metastasis to liver, wherein antibodies to αV/β3 were able to significantly reduce, but not completely suppress, tumor cell adhesion [19,28,37,39]. In addition, αV/β3 integrin expression alone was shown to enable non-tumor CHO cells to adhere and invade into to the liver [30,32,40,41]. These results suggest that inhibitors of integrin αV/β3 may be potent therapies for prevention of metastases. Though clinical trials of the monoclonal antibody to integrin αV, intetumumab, have shown limited success with metastatic disease, trials of this agent in patients with early-stage tumors for prevention has not been carried out [36,38,41,44]. The ABMEM demonstrated that integrin αV/β3 is a potential target for tumor metastasis prevention, and suggests a novel use of a therapeutic agent undergoing clinical trials.

The finding of an apparent saturation point in the effect of inhibition of platelet activation pathways on tumor cell adhesion is an example of the multi-component and interaction-dependent phenomena that can be examined using agent-based modeling. This finding suggests a mechanism by which tumor cells are able to adhere directly to endothelial cells via integrins, thus producing metastases despite potent inhibition of platelet adhesion within *in vivo* models [68,87].

The ABMEM, as with all computational models, has limitations in its scope and the parameters included within its design. This model does not incorporate mechanisms not specifically related to adhesion or activation of the cell types. Mechanisms such as cell cycle and replication are not included as these occur in time scales well beyond the model timeframe. Energy consumption is also not incorporated as it is assumed to be un-constrained by the environment of oxygenated blood. In addition, the range of cell adhesion molecules expressed on various cell types is quite diverse and previously unknown interactions are rapidly being discovered. Many of these are currently only described in certain contexts, while others appear to play a lesser role in the dynamics in question. These mechanisms may be incorporated in future versions of the ABMEM if proven to be essential and within its scope. Finally, some of the biophysical properties of the cells are abstracted, such as flow rate and resulting shear stress incorporated as cell movement and degeneration of bonds over time. Biomechanical properties such as cell membrane deformity and adhesion forces between cells are not included as the model focuses on the functional effects of cell pathways rather than the specific properties of these parameters. Despite these limitations, the model accurately reflects the known mechanistic knowledge of circulating tumor cell adhesion, correlates with findings from biological models, and presents evidence to support anti-platelet therapies as anti-metastatic.

The ABMEM is a novel proof of concept model that provides insights into the dynamic processes resulting in metastasis. A potential extension of this model includes incorporation of information regarding tumor-specific cell adhesion molecules expression patterns. In addition, the model can be expanded to include the effects of early inflammation induced by neutrophils and adherent tumor cells on vascular endothelial disruption and subsequent extravasation. These effects may modulate the pre-metastatic niche and early metastatic tumor survival.

## Materials and methods

The *Materials & Methods* section consists of a description of the ABM’s development and a description of the simulation experiments carried out with the model. The development followed the general process described in the *Overview, Design Concepts, Details (ODD)* protocol of Grimm, et al., modified for agent-based modeling of biological systems. Additionally, the iterative nature of model development emphasizes the successive addition of features to match more detailed observed variables to improve the model’s representation of the biological system. The ABM was implemented using NetLogo 5.0, which can be obtained online at http://ccl.northwestern.edu/netlogo/[9]. Initial simulation experiments include testing the validity of the ABM to reproduce normal circulating neutrophil, platelet and vascular endothelial interactions. Subsequent experiments introduced tumor cell interactions with these populations to validate known behaviors, and finally the model was used to examine the effects of defects in platelet, neutrophil or tumor cell pathways.

### Overview of ABM architecture

The ABM represents a two-dimensional patch of vascular endothelium that has been split open and laid flat into a toroidal square grid. Immobile endothelial cell agents along with circulating neutrophils, platelets and tumor cells populate the grid. Grid spaces within the model provide locations for agents, and possess environmental variables representing concentration of cytokines, reactive oxygen species and soluble proteins. The ABM incorporates spatial effect to examine the relationships between agents rather than specific histologic details. This model can be viewed as a cellular interaction network. Initial cell population ratios are based on physiologic ratios of platelets to neutrophils. The agent types and their internal variables are listed in Table 1. These variables are abstractions of molecular pathways manipulated by agents using logic-based and simple algebraic rules. They are updated and the resulting commands executed by each agent with each time step of the model. This ABM utilizes 1 time step to represent 1 second in order to reflect the timescale of the cell-cell interactions observed *in vitro* and *in vivo*. The complete code of the model can be found online upon publication.

### Description of secreted mediators implemented as environmental variables

Much of cell-to-cell interactions occur through secretion and binding of mediators secreted into circulation. The ABM simulates this paracrine behavior through the use of environmental (“Patch”) variables representing thrombin, fibrin, ADP, MMP2, interleukin-8, and reactive oxygen species. These variables are listed in Table 1. They represent the amount of a given signaling molecule in a given area, affecting those cells within that grid space. These variables are “produced” by cellular agents acting on their specific rules, and “bound” by other agents acting on their set of rules. This results in increasing or decreasing levels of a given variable on a grid space with each time step. To simulate the spread of these mediators through circulation, the ABM utilizes NetLogo’s “Diffuse” function, which reduces the value of a given environmental variable by a set percentage and distributes this amount to neighboring spaces. These parameters are calibrated in relation to each other and the model’s timescale, and against the behavior of agents during calibration simulations. Their values are listed in the Additional file 1.

### Description of cell types implemented as agents

The ABM emphasizes the interactions between circulating cells and interactions with the endothelium in a time scale representative of cellular adhesion. These interactions are described below, and globally depicted in Figure 1.

### Description of each cell type

#### Vascular endothelial cells

Vascular endothelial cell functions in the ABM are heavily abstracted, focusing on their role in circulating cell adhesion and response to inflammatory mediators. They are non-motile, but interact through paracrine means via environmental variables.

Their rules include:

1. Activation resulting in increased binding ability (representing selectin expression) when stimulated by thrombin, IL-8 or reactive oxygen species [15,88,89].

2. Production of IL-8 and nitric oxide (NO) when activated [22,26,90].

3. Degradation of circulating thrombin by anti-thrombin 3. This is an abstraction of the degradation of thrombin by endothelial cells through multiple mechanisms, including activated protein C and tissue factor pathway inhibitor [91,92].

4. Dying in response to excess reactive oxygen species, thus releasing bound circulating cells [31,93].

5. Down-regulation of adhesion molecules in response to nitric oxide [90,94]

#### Platelets

Platelets play an important role in adhesion of circulating tumor cells to endothelium. They can bind to tumor cells during circulation, enhancing their binding to endothelial cells. In addition, platelets bound to activated endothelium can also bind circulating tumor cells. Activated platelets also produce thrombin, which trigger inactive platelets in a paracrine manner. At initialization, platelets are randomly distributed throughout the field in an inactive form.

Rules of circulation:

1. Platelets circulate quasi-randomly through the model at set rate.

2. Platelets circulating off the model-space are destroyed and replaced.

3. Platelets bound to a neutrophil or tumor cell will move with the target cell.

Rules of activation:

1. Platelets bind thrombin, resulting in activation of PAR-1 and PAR-4. The relative strengths of activation and rates of de-activation were calibrated based on *in vitro* studies [30,36,37,87].

2. Activation of PAR-1, PAR-4 or P2Y12 results in increased intra-cellular calcium levels, triggering production of thromboxane, which further activates integrin GpIIb. GpIIb is deactivated at a set rate over time [30,37,95-98].

3. Activation of PAR-4 induces ADP production and ADP-dependent P-selectin activation [40,99-103].

4. Calcium induces thrombin production through coagulation factors and collagen. This production partially offset by baseline thrombin degradation via activated protein C [104-107].

Rules of interaction with endothelium:

1. Platelets with sufficient activated GpIIb and/or P-selectin will bind to an endothelial cell in their patch if the endothelial cell is also sufficiently activated [46,108].

2. Binding with endothelial cells induces degradation of platelet P-selectin to represent circulatory shear forces [109-111].

Rules of interaction with neutrophils:

1. Platelets are assigned, upon creation, a given binding threshold representing the amount of activated P-selectin necessary for binding to neutrophil integrins [112-114]. These thresholds are normally distributed across the population and calibrated within the model relative to other cell types. Platelets with activated P-selectin above their set threshold will bind to circulating neutrophils activated above their given threshold. They will disengage if levels of activation of either surface protein decreases below given thresholds.

Rules of interaction with tumor cells:

1. Platelets are assigned, as above, a threshold for GpIIb/IIIa activation and P-selectin binding necessary for tumor cell binding. These molecules bind with tumor cells expressing sufficient levels of αV/β3 in a manner similar to platelet-neutrophil interactions [5,16,115].

#### Neutrophils

Neutrophils also play an important role in adhesion of circulating tumor cells to endothelium. They can bind to tumor cells during circulation, enhancing their binding to endothelial cells. In addition, neutrophils roll across and bind to inflamed endothelium. These bound neutrophils primed by exposure to inflammatory cytokines will produce reactive oxygen species, a processed termed “respiratory burst”. These neutrophils also release inflammatory cytokines and pro-coagulants, promoting further neutrophil and platelet activation. At initialization of the model, neutrophils are randomly distributed throughout the field in an inactive form.

Rules of circulation:

1. Neutrophils circulate quasi- randomly through the model at a set rate.

Rules of interaction with endothelium:

1. Neutrophils exposed to the inflammatory cytokine IL-8 will be “primed”, activating L-selectin and N-cadherin, rolling along endothelial cells at a slow speed [40,99,101-103]. L-selectin is decreased as the neutrophil rolls to represent shedding of this molecule, which diminishes further neutrophil adhesion by competitive inhibition [116,117].

2. Primed neutrophils will also activate Macrophage-1 antigen (Mac-1 or integrin αM/β2) in response to IL-8 [23,47,118-120]. Rolling neutrophils with sufficient activation will bind to an endothelial cell in their patch if the endothelial cell is also sufficiently activated [121,122].

3. Neutrophil activation of adhesion molecules is inhibited by nitric oxide [109,111].

Rules for interaction with tumor cells:

1. Neutrophils are assigned a threshold for binding to tumor cells representing activation of Mac-1 and Lymphocyte-Function Associated Antigen 1 (LFA-1 or integrin αL/β2). Sufficiently activate neutrophils bind tumor cells through αV/β3 integrin and ICAM-1, also represented by an activation threshold [49,119,123]. This is calibrated in relation to other cell-cell interactions within the model.

Rules for interaction with platelets:

1. Neutrophils will bind to platelets in a reciprocal relation described above.

2. Binding to platelets via MAC-1 and LFA-1 (integrins αM/β2 and αL/β2) contribute to priming for oxidative burst [124-126].

Rules for activation of oxidative burst:

1. Neutrophils exposed to IL-8 or thrombin and bound to other cell types through β2 integrin will become progressively more activated, thus increasing both integrin expression and reactive oxygen species [99,120,127-129].

2. This process is partially inhibited by endothelial cells’ release of nitric oxide [130].

3. Once sufficiently activated above a set threshold, the neutrophil will produce reactive oxygen species until deactivated or killed from oxidative damage at a set threshold.

4. ROS production is capable of killing tumor cells and endothelial cells if the local concentration exceeds set thresholds for each cell type. These thresholds were set to a level that resulted in death of cell types over the timescale of *in vitro* and *in vivo* experiments (30 minutes to 4 hours) [116,130-133].

#### Tumor cells

Tumor cells in this simulation possess abstract representations of the major cell surface molecules experimentally associated with endothelial, platelet and neutrophil adhesion. Though modeled on melanoma, breast and colorectal tumor cells, they are generalizable to most tumor types. They also constitutively produce inflammatory cytokines, represented by IL-8, and induce thrombin generation. The specific internal details of the tumor cells are not modeled. At initialization of the model, tumor cells are randomly distributed throughout the field in an inactive form.

Rules of circulation:

1. Tumor cells circulate quasi- randomly through the model at a set rate.

Cytokine production:

1. Metastatic tumor cells produce interleukin-8, which activates PMNs, platelets and endothelial cells as described above [50,121,134-136].

2. Tumor cells induce thrombin generation *S*, which activates platelets [23,27,45,34,137-141].

Rules of interaction with endothelium:

1. Tumor cells are able to bind to endothelium in a similar manner to platelets, through integrin β2 and selectins, represented in aggregate within the model [40,60,142].

Rules for interaction with neutrophils and platelets:

1. Tumor cells bind with neutrophils and platelets in reciprocal interactions as described above.

Process of Fitting and Calibration:

Each cell adhesion molecule is controlled by two sets of rules governing the activation of the molecules and the thresholds necessary for effective binding. To determine the optimal parameters for replicating experimentally observed behaviors between cell types, a range of values for each threshold was inputted (parameter sweeping), and effects on cell-cell adhesion in the time frame used in the reference experiments were quantified. These parameters were then adjusted to qualitatively replicate referenced results. Criteria for fitting included 1) replicating the observed adhesion interactions in the time frame observed experimentally and 2) replicating the effects of inhibition of each molecular component of the cell-cell interaction. Refer to Additional file 1 for examples of model calibration. Adjustment of parameters over a broad range of values resulted in minor changes in quantitative effects (example in Additional file 1: Figure S4).

## Conclusions

The Agent-Based Model of Early Metastasis (ABMEM) provides an effective platform for hypothesis generation concerning the early events of metastasis formation, namely the initial adhesion of circulating tumor cell complexes in the target organs, and examination of potential anti-metastatic therapies. It allows for dynamic representation of current mechanistic knowledge of complex tumor cell interactions with the host environment. The ABMEM suggests that inhibition of platelet mechanisms of activation, mediated by thromboxane and ADP receptors, may strongly inhibit stable circulating tumor cell adhesion. In addition, the ABMEM suggests a novel use of inhibiting circulating tumor cell Integrin αV/β3 to also prevent stable adhesion to the endothelium. Inhibition of stable tumor cell adhesion, a critical step in the metastatic cascade, may lead to novel anti-metastasis therapies. These findings demonstrate the utility of Agent-Based Modeling in understanding phenomena that are difficult to examine with traditional experimental methods.

## Abbreviations

ABM: Agent Based Model; ABMEM: Agent Based Model of Early Metastasis; ADP: Denosine Diphosphate; IL-8: Interleukin 8; NO: Nitric Oxide.

## Competing interests

The authors declare that they have no competing interests.

## Authors’ contributions

AU and GA conceived of and participated in the design of the model, and helped draft the manuscript. SCW, SG and RRW participated in the design of the model, and helped revise the manuscript. All authors read and approved the final manuscript.

## Supplementary Material

Additional file 1: Figure S1 Calibration of Neutrophils binding to endothelium. **Figure S2.** Calibration of Platelet Binding to Neutrophils in circulation. **Figure S3.** Calibration of tumor cell binding to platelets in circulation. **Figure S4.** Representative parameter sweeps. Click here for file
