# Supplementary material for: Investigation of the essential role of platelet-tumor cell interactions in metastasis progression using an agent-based model
Source: Theor Biol Med Model. 2014 Apr 12;11:17. doi: 10.1186/1742-4682-11-17 (PMC4022382; doi:10.1186/1742-4682-11-17)
Supplement: Additional file 1: Figure S1 — Calibration of Neutrophils binding to endothelium. Figure S2. Calibration of Platelet Binding to Neutrophils in circulation. Figure S3. Calibration of tumor cell binding to platelets in circulation. Figure S4. Representative parameter sweeps. [file 1742-4682-11-17-S1.pdf]

Additional File 1

Figure S1. Calibration of Neutrophils binding to endothelium. Neutrophils' binding thresholds for MAC-1 and N-cadherin were adjusted to achieve at least 70% of tumor cell binding to activated neutrophils within 300 ticks. Panel A: Decreased adhesion of neutrophils to endothelial cell monolayer *in vitro* after inhibition of neutrophil cell surface receptors Integrin β2 or N-cadherin with monoclonal antibodies. Panel B: Inhibition of these the β2 or N-cadherin surface receptors in the neutrophil agents of the ABMEM results in similar proportional reduction in neutrophils adhered to endothelial agents. β2 = antibody to integrin β2, N-cad = antibody to N-cadherin. * = $p<0.01$, ** = $p<0.001$.

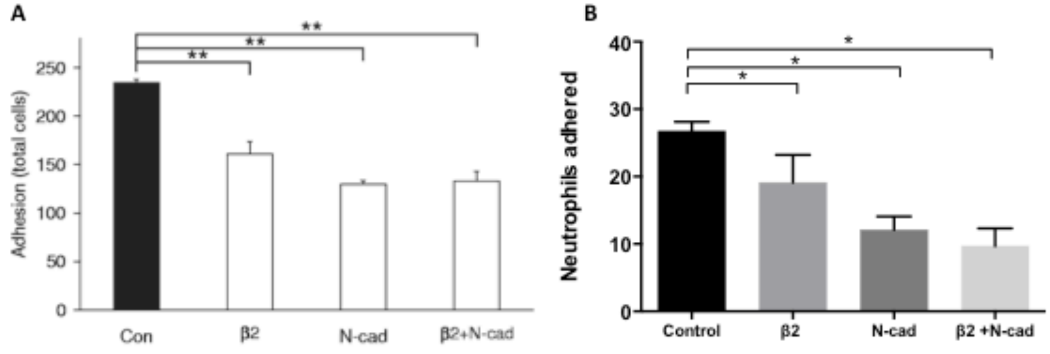

Panel A reprinted with permission from: Strell C, Lang K, Niggemann B, Zaenker KS, Entschladen F: **Surface molecules regulating rolling and adhesion to endothelium of neutrophil granulocytes and MDA-MB-468 breast carcinoma cells and their interaction.** *Cell Mol Life Sci* 2007, **64**:3306–3316.

Figure S2. Calibration of Platelet Binding to Neutrophils in circulation.

Platelets' binding thresholds for P-selectin and neutrophils' binding thresholds for MAC-1 were adjusted to replicate experimentally observed time-courses for platelet adhesion to neutrophils within the ABMEM. Panel A: Effect of blocking of platelet P-selectin, Neutrophil Mac-1 (CD11b/18) or both on number of platelets adhered to neutrophils, measured as fluorescent intensity of bound platelets relative to neutrophils. fMLP/Thrombin timecourse represents uninhibited, activated platelets in the presence of thrombin and fMLP. All other experiments were carried out in the presence of fMLP and Thrombin. Panel B: Timecourse of platelet adhesion to neutrophils in the ABMEM demonstrating a similar time range for binding of activated platelets to neutrophils, and similar suppression of binding with inhibition of P-selectin or Mac-1.

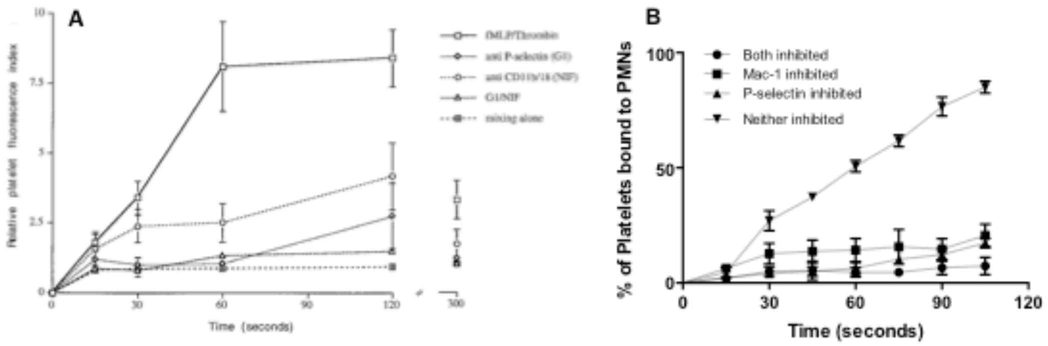

Panel A reprinted with permission from: Brown KK, Henson PM, Maclouf J, Moyle M, Ely JA, Worthen GS: **Neutrophil-platelet adhesion: relative roles of platelet P-selectin and neutrophil β2 (DC18) integrins.** *Am J Respir Cell Mol Biol* 1998, **18**:100–110. Copyright American Thoracic Society, 2013

Figure S3. Calibration of tumor cell binding to platelets in circulation. Platelets' binding thresholds for activated P-selectin and GpIIb/IIIa in the presence of thrombin were adjusted to replicate experimentally observed decreased in adhesion to tumor cells after selective inhibition. Panel A: Percentage of A375 melanoma cells adherent to a platelet monolayer is decreased after blocking of platelet integrin GpIIb/IIIa, but not P-selectin. 9E1 = monoclonal antibody to P-selectin, 10E5 = monoclonal antibody to Integrin GpIIb/IIIa. Panel B: Tumor cell adhesion to platelet agents in the ABMEM is decreased by inhibition of platelet integrin GpIIb/IIIa, but is not affected by inhibition of P-selectin. *, $p < 0.05$; ** $p < 0.01$.

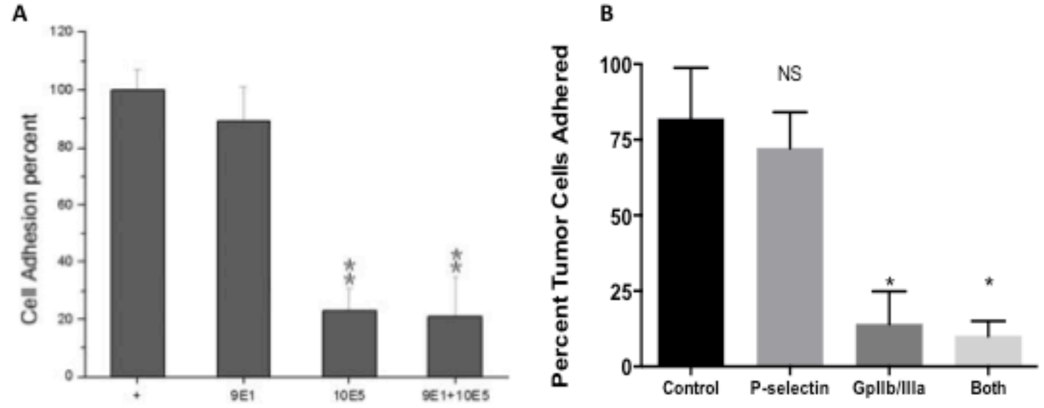

Panel A reprinted with permission from: Zhang C, Liu Y, Gao Y, Shen J, Zheng S, Wei M, Zeng X: **Modified heparins inhibit integrin αIIb/β3 mediated adhesion of melanoma cells to platelets in vitro and in vivo**. *J Pathol* 2009, **125**:2058–2065.

Figure S4. Representative parameter sweeps. Adjustment of the endothelium-binding threshold for neutrophils, a variable controlling the level of integrin β2 and N-cadherin binding necessary for adhesion, resulted in minor variations in the number of bound cells across a wide range of values. Similar results were obtained for parameter sweeps of other adhesion variables, indicating that the model is insensitive to minor changes in threshold values. PMN = neutrophils, EC = endothelial cells.

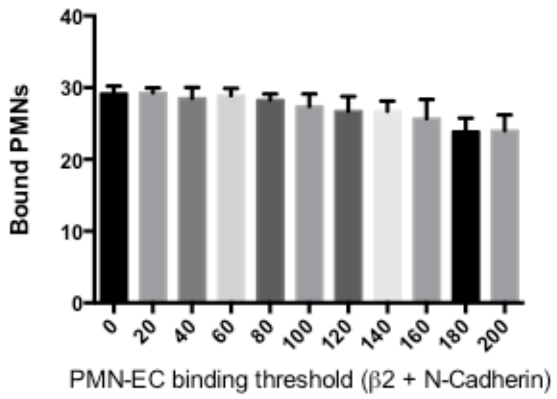

Calibration of tumor cell binding to Neutrophils in circulation. Tumor cells' binding thresholds for activated αv/β3 and neutrophil binding thresholds for MAC-1 and LFA-1 in the presence of thrombin were adjusted to achieve at least 70% of tumor cell binding to activated neutrophils within 300 seconds of simulated time, as compared to Zhang P, Ozdemir T, Chung C-Y, Robertson GP, Dong C: Sequential binding of αV/β3 and ICAM-1 determines fibrin-mediated melanoma capture and stable adhesion to CD11b/CD18 on neutrophils. *J Immunol* 2011, 186:242–254.
